# Effect of β-blockade on measures and reproducibility of heart rate, oxygen uptake and work rate across repeated bouts of short-duration, RPE-regulated exercise

**DOI:** 10.1007/s00421-025-05818-x

**Published:** 2025-06-01

**Authors:** Braden L. Mitchell, Kade Davison, Gaynor Parfitt, Roger G. Eston

**Affiliations:** Alliance for Research in Exercise Nutrition and Activity (ARENA), University of South Australia, Adelaide, SA Australia

**Keywords:** Rating of perceived exertion, Beta-blockade, Exercise intensity, Perceptual regulation, Perceptually regulated exercise, Interval training

## Abstract

**Purpose:**

We examined the effect of β-blockade on measures and reproducibility of heart rate (HR), oxygen uptake (${\dot{\rm{V}}}{\rm{O}}_{2}$) and work rate (WR) across repeated bouts of short-duration, RPE-regulated exercise.

**Methods:**

Participants completed an RPE-regulated, interval-based exercise session under control and β-blockade conditions with six 3-min bouts alternating between RPE 13 and RPE 15, separated by 2-min active recovery periods. Participants adjusted treadmill speed/grade to meet the target RPE. Linear mixed effects models assessed the effect β-blockade on exercise responses for each RPE, while intraclass correlation coefficients (ICC) and coefficients of variation (CV) evaluated reproducibility across bouts.

**Results:**

β-Blockade significantly reduced HR (− 36.5 beat min^−1^, *p* < 0.001), ${\dot{\rm{V}}}{\rm{O}}_{2}$ (− 4.2 mL kg^−1^ min^−1^, *p* < 0.001) and work rate (− 0.6 METs, *p* = 0.022) during exercise. Differences between conditions remained significant for %HR_peak_ (*p* < 0.001) but not %${\dot{\rm{V}}}$O_2peak_ or %WR_peak_ (*p* > 0.05). Exercise responses were consistently higher at RPE 15 than RPE 13 (all *p* < 0.001). A significant interaction showed greater HR reduction at RPE 15 (45.5 beat min^−1^) than RPE 13 (40.0 beat min^−1^) under β-blockade (*p* = 0.041). ICC values indicated good to excellent reproducibility across bouts, with no significant difference between conditions. Variability across bouts was low (mean CV = 2–8%) and unaffected by β-blockade.

**Conclusion:**

Our findings suggest that despite significant reductions in absolute responses, β-blockade does not affect relative measures of ${\dot{\rm{V}}}$O_2_ or work rate. RPE-regulated exercise may facilitate highly reproducible exercise intensities, making it particularly valuable for populations where β-blocker use is prevalent.

**Supplementary Information:**

The online version contains supplementary material available at 10.1007/s00421-025-05818-x.

## Introduction

Regulating exercise intensity is fundamental to effective exercise prescription, with direct implications for safety, adherence and training outcomes (ACSM 2018). While physiological indices of heart rate or oxygen uptake (${\dot{\rm{V}}}$O_2_) are commonly used to prescribe exercise intensity, their utility in regulating intensity during exercise has limitations in certain contexts. The rating of perceived exertion (RPE) represents a practical and widely accessible alternative for both prescribing and regulating exercise intensity.

When used to regulate intensity directly, individuals adjust their effort in real time to match their interpretation of a prescribed RPE level, facilitating a self-paced but structured form of training. A growing body of evidence supports the use of RPE as a primary method for regulating exercise intensity (Eston and Parfitt 2019), with training studies demonstrating that RPE-regulated exercise elicits similar improvements in cardiorespiratory fitness to those achieved through conventional heart rate-based methods (Parfitt et al. 2012, 2015).

Despite its ubiquitous use to regulate exercise intensity across a variety of athletic, recreational and pedagogical settings, heart rate presents a challenge when applied in clinical contexts where cardiac function is altered by disease or pharmacological treatment—most notably with β-blockers. Frequently prescribed during early cardiac rehabilitation and in the management of heart failure and arrhythmia (Chew et al. 2016; López-Sendón et al. 2004), these medications complicate exercise prescription by lowering heart rate across the entire intensity spectrum (Van Baak 1988; Wilmore et al. 1985) and disrupting the typical heart rate–${\dot{\rm{V}}}$O_2_ relationship. While the relative relationship between measures may remain stable within individuals (Eston and Connolly 1996; Gordon and Duncan 1991; Mitchell et al. 2019; Van Baak 1988), maximal heart rate can no longer be accurately estimated, and heart rate zones used to define moderate or vigorous intensities (ACSM 2018) may no longer correspond to actual physiological effort, limiting the practical utility of this approach. Furthermore, any changes in medication regimen or compliance may significantly alter heart rate responses, further undermining the reliability of heart rate-based methods.

RPE-regulated exercise provides a viable alternative, offering a practical method for individuals to self-regulate exercise intensity. However, it is unclear whether RPE remains a valid and reliable means of regulating exercise intensity during β-blockade. This uncertainty arises from the wide-ranging physiological effects of β-blockade, which not only modify central cardiovascular function but also influence multiple systems involved in the perception of effort. In addition to its primary therapeutic effects in reducing heart rate, cardiac output, blood pressure, and myocardial oxygen demand (Gordon and Duncan 1991; Van Baak 1988; Wilmore et al. 1985), β-blockade also alters a range of systemic responses during exercise, including ventilatory and metabolic function, peripheral circulation and oxygen extraction, and thermoregulation (Jilka et al. 1988; Joyner et al. 1987, 1986; Tesch and Kaiser 1983; Wilmore et al. 1985). These alterations occur alongside reductions in both maximal ${\dot{\rm{V}}}$O_2_ and submaximal ${\dot{\rm{V}}}$O_2_ at a given work rate (Ekblom and Goldbarg 1971; Mitchell et al. 2019; Wilmore et al. 1985)—though whether such reductions persist with long-term β-blockade remains uncertain.

The integration of physiological signals into the perceived exertion gestalt remains poorly understood, making it difficult to predict how changes induced by β-blockade may influence perceptions of effort. Whether β-blockade affects RPE directly—through altered perceptual or central integrative processes—or indirectly via its effects on cardiovascular, respiratory, and metabolic responses, remains unclear, as does the extent to which such changes may compromise the validity and reliability of RPE to regulate exercise intensity. Previous research suggests that RPE is less affected by β-blockade than heart rate, and that observed changes in RPE tend to correspond more closely with reductions in ${\dot{\rm{V}}}$O_2_ (Davies and Sargeant 1979; Ekblom and Goldbarg 1971; Eston and Connolly 1996; Mitchell et al. 2019). However, these studies have exclusively employed RPE within an estimation paradigm, in which participants passively rate their perceived effort during externally imposed exercise intensities. This contrasts with production paradigms—such as RPE-regulated exercise—where individuals actively adjust their effort to match a prescribed level of exertion, a process considered by some to be psycho-physiologically distinct (Noble 1982). This distinction challenges the assumption that findings from estimation paradigms generalise to RPE-regulated exercise, highlighting the need to directly examine whether RPE remains valid and reliable for regulating intensity under β-blockade.

This study aimed to examine the effect of β-blockade on the validity and reproducibility of exercise responses across repeated bouts of short-duration, RPE-regulated exercise. Participants completed an interval-based protocol under control and β-blockade conditions, self-regulating intensity to RPE 13 and RPE 15. Findings will inform the utility of RPE-regulated exercise in populations for whom β-blocker use is common. We hypothesised that: (a) at a given RPE level, heart rate would be lower under β-blockade, while ${\dot{\rm{V}}}$O₂ and work rate would remain consistent across conditions; (b) when standardised to within-condition peak measures, heart rate, ${\dot{\rm{V}}}$O₂ and work rate would be comparable across conditions; and (c) the reproducibility of heart rate, ${\dot{\rm{V}}}$O₂ and work rate would be unaffected by β-blockade.

## Methods

### Participants

Sixteen participants were recruited from the staff and student populations of local university campuses. Recruitment involved a screening questionnaire, where participants self-reported as non-smokers, normotensive (90–140/60–85 mmHg), non-bradycardic (≥ 45 beat min^−1^ at rest) and not taking prescribed medications (excluding contraceptives). Individuals with a history of asthma, current cardiac arrhythmia, cardiovascular, liver or metabolic diseases, or those with an allergy requiring exogenous epinephrine were excluded.

All participants provided written informed consent and obtained medical clearance before participation. Ethical approval for the study was provided by the University of South Australia Human Research Ethics Committee.

### Procedures

Data presented here are part of a larger study investigating the effects of β-blockade on effort perception during exercise that utilised a double-blind, placebo-controlled, randomised and counter-balanced design. Participants in the larger study attended six sessions. In the first four sessions, they completed a submaximal perceptually regulated exercise test (sessions 1, 3) and a maximal GXT (session 2, 4) on separate days. Sessions were randomised in pairs to be completed under control or β-blockade conditions. Participants were then re-randomised to complete an interval-based, RPE-regulated exercise session under each condition (sessions 5, 6). This analysis focuses on data obtained during the maximal GXT and interval-based exercise sessions.

A 100 mg dose of the oral β_1_-selective antagonist metoprolol tartrate (Lopressor 100; Novartis AG, Basel Switzerland) was used for the β-blockade condition, while a lactose placebo of equivalent weight served as the control. Metoprolol tartrate was selected as it is commonly prescribed for patients following myocardial infarction and revascularisation procedures in the local public hospital system where this study was conducted. To ensure blinding, both the β-blockade and control conditions were encapsulated in identical dark blue, opaque capsules, prepared by an experienced pharmaceutical technician at the University of South Australia. Research personnel not directly involved in data collection accessed the randomisation prior to each session, placing the assigned capsule in a sealed envelope so neither the participant nor researcher conducting the session were explicitly aware of the condition.

Participants were instructed to fast for a minimum of 2 h prior to each session to standardise absorption of the β-blocking agent. They were also required to abstain from caffeine for at least 6 h, and alcohol and vigorous exercise for at least 12 h. Participants were advised to wear comfortable, loose-fitting clothing and appropriate footwear for treadmill running, avoiding compression garments. The researcher confirmed compliance with these requirements verbally at the beginning of each session.

On arrival, heart rate and blood pressure were measured following a 5-min seated rest. Participants then ingested the assigned capsule, with heart rate and blood pressure monitored every 15 min thereafter. Exercise testing commenced 60–90 min post-ingestion consistent with pharmacokinetic studies indicating metoprolol tartrate reaches peak plasma concentrations ~ 60 min after ingestion (Regårdh et al. 1974). A washout period of at least 72 h was used to accommodate participants who may exhibit the CYP2D6 ‘poor metaboliser’ phenotype; approximately 10% of the Caucasian population (Lennard et al. 1982; Rau et al. 2002).

All testing sessions were conducted on a motorised treadmill (JT4000; JNB Sports) within a temperature-controlled laboratory maintained at 21 °C. The maximal GXT commenced at 4.0 km h^−1^ and 1% grade, with increments of 2.0 km h^−1^ every 2 min. Tests were terminated upon the participant’s volitional exhaustion, despite receiving verbal encouragement throughout.

**Fig. 1:**
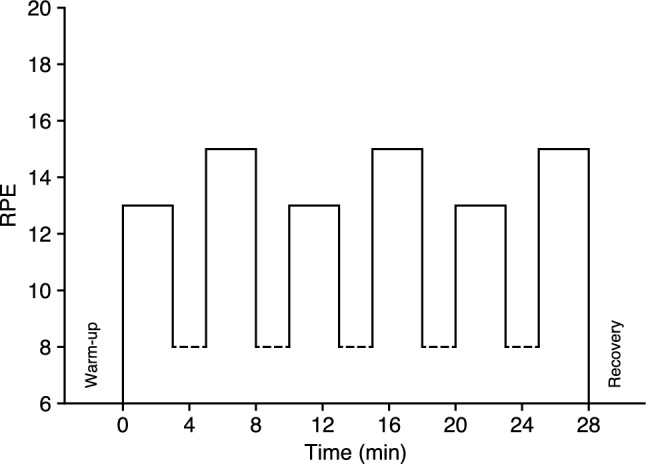
Protocol for the perceptually regulated, interval-based exercise sessions. Dashed lines represent active recovery periods that were not perceptually regulated; corresponding RPE values are illustrative only

The protocol for the RPE-regulated interval session, illustrated in Fig. 1, included six 3-min bouts of exercise at alternating intensities of RPE 13 and RPE 15 (Borg 6–20 RPE scale), interleaved with 2-min active recovery periods. We selected these RPE targets to reflect moderate and vigorous exercise intensities, as recommended by the ACSM (2018, p. 146), with studies from our lab demonstrating the efficacy of long-term exercise training at these RPE levels to improve cardiorespiratory fitness (Parfitt et al. 2012, 2015; Evans et al. 2013). The 3-min bouts allowed participants adequate time to adjust their work rate to reach the target RPE and is consistent with bout length in our previous studies on perceptually regulated exercise testing (e.g., Eston et al. 2012). This also kept the total exercise duration under 30 min to minimise any potential impact of fatigue in the later stages of the session.

Each session began with a 2-min warm-up at 4.0 km h^−1^ and 1% grade. At the start of each bout, participants adjusted treadmill speed and/or grade to reach the target RPE and made further adjustments to their work rate as needed throughout to fine-tune their level of exertion. We chose to allow both speed and grade adjustments to give participants complete control and better reflect how they would naturally regulate their exertion. The treadmill provided increments of 0.1 km h^−1^ and 1%. Treadmill grade was restricted to a minimum of 1% to parallel the oxygen cost associated with over-ground running (Jones and Doust 1996), though participants could increase from this as needed. Participants confirmed their RPE each minute and were reminded of the target RPE if this varied. Between bouts, the treadmill returned to 4.0 km h^−1^ and 1% grade for active recovery, ensuring a sufficiently low intensity to reduce heart rate and ${\dot{\rm{V}}}$O_2_, while also providing a consistent starting point for each bout.

Prior to each RPE-regulated session, participants received standardised verbal instructions to anchor their perception of effort using the Borg 6–20 RPE scale within an effort-production paradigm. Delivered as part of the explanation of the interval protocol, these instructions followed a set script to ensure consistency across participants. The script, provided in the Online Resource, emphasised that RPE should reflect their overall perception of effort, incorporating all exercise sensations such as muscular strain and fatigue, breathlessness and chest tightness. Participants were instructed to adjust treadmill speed and/or grade as needed to match the target RPE using their perception of their current effort rather than external cues. After receiving the instructions, participants had the opportunity to seek clarification of the scale if needed.

Importantly, the treadmill display was concealed during all exercise sessions using an A4 copy of the Borg 6–20 RPE scale. This masked the speed and grade settings from participants, along with information regarding exercise distance and duration, while still allowing access to the controls. Similarly, all physiological data were hidden from view. This setup ensured participants relied exclusively on real-time exercise sensations to regulate their work rate, thereby eliminating any potential influence of recall from the current testing session, as well as any previous testing sessions or treadmill experiences. Consequently, participants were also not informed of the specific settings for the warm-up and active recovery periods, nor were they made aware of their performance in any sessions until completion of the study.

### Measures

Breath-by-breath measures of pulmonary ventilation and gas exchange were collected via metabolic cart (MetaLyzer 3B-R2; Cortex Biophysik, Leipzig, Germany) and continuous heart rate data were recorded using a wireless heart rate monitor (RS800cx; Polar Electro, Kempele, Finland). The heart rate data were filtered using the supplied software (ProTrainer v5.4) to eliminate artefacts from the R–R signal and subsequently converted to units of beats per minute (beat min^−1^). The ventilation, gas exchange and heart rate data were then combined and exported in 10-s retrospective block averages for analyses.

As participants could adjust both the speed and grade of the treadmill, we estimated oxygen uptake requirements as an indicator of selected work rates. These estimates were calculated from the treadmill speed and grade at the end of each bout using the metabolic equation for treadmill running (ACSM 2018, p. 152). The running equation was used since all participants selected speeds that necessitated running during each bout. The resulting values are expressed in metabolic equivalents (METs) to avoid confusion with objective oxygen uptake measures.

Peak oxygen uptake (${\dot{\rm{V}}}$O_2peak_) and heart rate (HR_peak_) were defined as the highest 30-s rolling average and highest 10-s average, respectively, from the maximal GXT for each condition and peak work rate (WR_peak_) was defined as the highest estimated METs achieved. Ventilatory threshold was determined using the modified V-slope method (Gaskill et al. 2001), with ${\dot{\rm{V}}}$O_2_ and heart rate at the deflection point recorded as the values corresponding to the ventilatory threshold.

### Statistical analyses

The primary outcome measures included heart rate, ${\dot{\rm{V}}}$O_2_ and work rate at the end of each bout. Values for heart rate were taken as the mean over the final ten seconds of each bout, and for ${\dot{\rm{V}}}$O_2_ as the mean over the final 30 s. Work rate was taken as the estimated METs derived from the final speed and grade for each bout as described previously.

Linear mixed effects models assessed the effects of β-blockade and RPE level on exercise responses. Separate models were constructed for each outcome, with values also expressed relative to their within-condition peak from the maximal GXT. Models included fixed effects for condition (control, β-blockade), RPE level (RPE 13, RPE 15) and exercise bout. Initially, we fitted fully specified models with all first- and second-order interactions; however, likelihood ratio tests showed the second-order interaction did not significantly improve model fit, thus we opted to present the simpler models. Exercise bout was treated as a continuous discrete variable, ranging from 1 to 3, reflecting the order of bouts within each RPE level. Participants were included as a random effect to account for the repeated measures design, and models were fit using restricted maximum likelihood estimation due to the limited sample. We report the estimated fixed effects, 95% confidence intervals (CI) and associated *p*-values obtained via Satterthwaite’s method for approximating degrees of freedom. Pairwise comparisons were used to further examine significant interactions in the models, alongside planned contrasts to assess differences between conditions within each RPE level. Model assumptions were assessed using diagnostic plots of residual vs. fitted values for signs of heteroscedasticity and Q–Q plots for normality of residuals.

Intraclass correlation coefficients (ICC) examined the effect of β-blockade on reproducibility of exercise responses across bouts. The ICCs were calculated using a two-way, mixed-effects model with an absolute agreement definition for single measures (ICC_3,1_) and interpreted per the recommendations of Koo and Li (2000). Fisher’s *z*-transformation was applied, allowing *p*-values for the difference in *z*-scores between conditions to be derived from the cumulative distribution function of the standard normal distribution.

Coefficients of variation (CV) were used to quantify individual participant variability in exercise responses across bouts. Coefficients were calculated separately for each condition and RPE level as the ratio of the standard deviation to the mean for each participant. Repeated measures ANOVA was used to examine the effect of β-blockade on variability in exercise responses.

Analyses were performed in R (version 4.4.1), using the *lme4* package (version 1.1-35) for modelling, *emmeans* package (version 1.10.5) for contrasts and *irr* package (version 0.84.1) to calculate ICCs. Significance was set at *α* = 0.05.

## Results

Data from the two interval sessions were unavailable for three participants due to withdrawal from the study (*n* = 2) and an unrelated injury (*n* = 1). Table 1 presents the characteristics of the 13 participants included in this analysis. Differences between conditions in heart rate, ${\dot{\rm{V}}}$O_2_ and work rate from the maximal GXT are also provided in Table 1 and have been examined in greater detail previously (Mitchell et al. 2019). Additionally, Table 1 includes exercise responses associated with RPE 13 and RPE 15 during the maximal GXT.

**Table 1:** Sample demographics, resting cardiovascular measures, and cardiopulmonary and work rate responses during graded exercise testing, with interpolated values for RPE 13 and RPE 15

	Control	β-Blockade
*n* (men)	13 (7)	
Age, year	25.8 ± 4.8	
Height, cm	170.4 ± 9.6	
Body mass, kg	65.4 ± 10.5	
BMI, kg m^−2^	22.4 ± 1.9	
Rest		
Heart rate, beat min^−1^	59.7 ± 7.0	50.8 ± 5.7*
Systolic BP, mmHg	112.1 ± 5.1	107.0 ± 7.0*
Diastolic BP, mmHg	67.8 ± 6.6	66.1 ± 7.4
SpO_2_, %	98.3 ± 0.7	98.3 ± 0.5
Graded exercise testing		
HR_vt_, beat min^−1^	136.0 ± 12.3	109.6 ± 9.9*
HR_peak_, beat min^−1^	190.8 ± 7.8	153.5 ± 22.2*
${\dot{\rm{V}}}$O_2vt_, mL kg^−1^ min^−1^	30.0 ± 3.6	28.5 ± 4.2*
${\dot{\rm{V}}}$O_2max_, mL kg^−1^ min^−1^	51.1 ± 5.8	47.8 ± 7.6*
WR_peak_, METs	17.2 ± 1.8	16.3 ± 2.4*
Responses at RPE 13		
Heart rate, beat min^−1^	145.7 ± 11.8	112.4 ± 13.9*
%HR_peak_	76.4 ± 6.0	73.6 ± 6.0*
${\dot{\rm{V}}}$O_2_, mL kg^−1^ min^−1^	31.0 ± 5.4	27.5 ± 4.8*
%${\dot{\rm{V}}}$O_2max_	61.0 ± 9.8	57.9 ± 8.3
Work rate, METs	10.4 ± 1.4	9.7 ± 1.2
%WR_peak_	66.2 ± 9.2	65.0 ± 7.8
Responses at RPE 15		
Heart rate, beat min^−1^	163.6 ± 10.7	126.1 ± 16.1*
%HR_peak_	85.8 ± 5.5	82.4 ± 5.1*
${\dot{\rm{V}}}$O_2_, mL kg^−1^ min^−1^	37.7 ± 5.5	34.0 ± 5.0*
%${\dot{\rm{V}}}$O_2max_	74.0 ± 8.8	71.7 ± 7.1
Work rate, METs	12.3 ± 1.6	11.5 ± 1.3*
%WR_peak_	78.2 ± 9.1	76.8 ± 6.7

*BMI* body mass index, *BP* blood pressure, *SpO*_*2*_ oxygen saturation, *HR*_*vt*_ heart rate at ventilatory threshold, *HR*_*peak*_ peak heart rate, ${\dot{{V}}}$*O*_*2vt*_ oxygen uptake at ventilatory threshold, ${\dot{V}}$*O*_*2max*_ maximal oxygen uptake, *WR*_*peak*_ peak work rate

*p < 0.05 vs. control

**Fig. 2:**
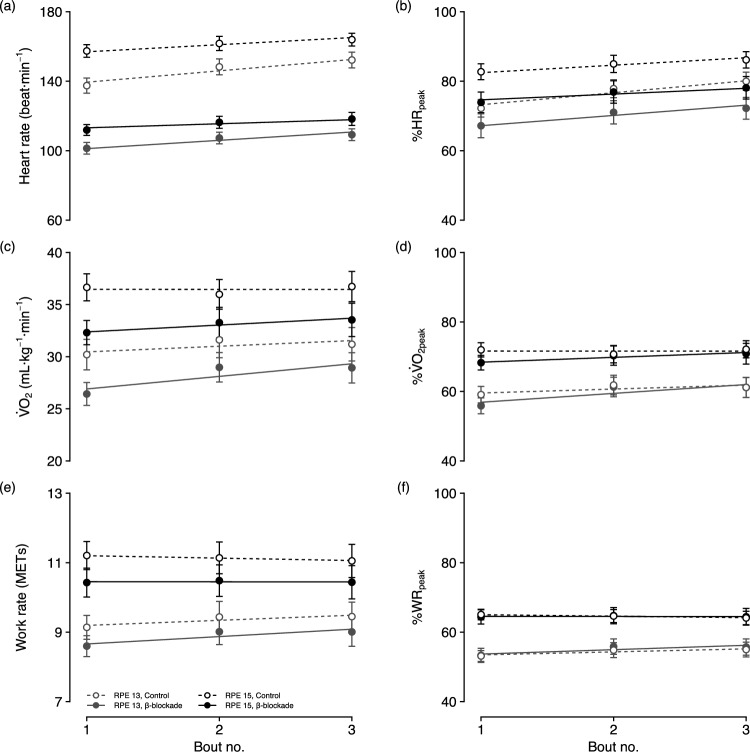
Linear mixed effects models for heart rate (top), oxygen uptake (middle) and work rate (bottom) across repeated bouts of perceptually regulated exercise, by condition and RPE level. Absolute values are shown on the left and relative to within-condition peak on the right. Error bars represent mean ± SE for each bout

The effect of β-blockade on heart rate, ${\dot{\rm{V}}}$O_2_ and work rate during the RPE-regulated, interval-based exercise session is illustrated in Fig. 2. Full outputs and diagnostic plots for the linear mixed effects models are provided in the Online Resource. Models indicated that participants exercised at higher work rates for RPE 15 compared to RPE 13 (β [95% CI] = 2.22 [1.72, 2.72], *p* < 0.001), along with increased heart rates (19.94 [12.64, 27.23], *p* < 0.001) and ${\dot{\rm{V}}}$O_2_ (6.55 [4.79, 8.32], *p* < 0.001). Additionally, β-blockade significantly reduced heart rate (− 36.49 [− 43.79, − 29.20], *p* < 0.001), ${\dot{\rm{V}}}$O_2_ (− 4.22 [− 5.99, − 2.49], *p* < 0.001) and work rate (− 0.60 [− 1.10, − 0.10], *p* = 0.022) during exercise compared to the control condition. The effects of RPE level and condition remained significant for both %HR_peak_ (10.53 [6.54, 14.52], *p* < 0.001; − 5.55 [− 9.54, − 1.56], *p* = 0.008) and %${\dot{\rm{V}}}$O_2peak_ (13.24 [9.48, 17.01], *p* < 0.001; − 4.07 [− 7.84, − 0.30], *p* = 0.039); however, only the effect for RPE level remained significant for %WR_peak_ (12.86 [9.82, 15.90], *p* < 0.001). Significant increases in both absolute (6.54 [3.80, 9.37], *p* < 0.001) and relative (3.45 [1.95, 4.94], *p* < 0.001) heart rates were observed across bouts, whereas no significant changes were noted in ${\dot{\rm{V}}}$O_2_ (*p* = 0.116), work rate (*p* = 0.133), or their relative indices (*p* > 0.10).

A significant condition × RPE level interaction was identified for heart rate only (− 5.52 [− 10.68, − 0.36], *p* = 0.041). Pairwise comparisons showed that β-blockade produced a larger reduction in heart rate at RPE 15 (45.5 beat min^−1^) than at RPE 13 (40.0 beat min^−1^) and attenuated the increase in heart rate between RPE levels (9.5 beat min^−1^) relative to control (15.1 beat min^−1^). No other significant interactions were observed in any of the models.

**Fig. 3:**
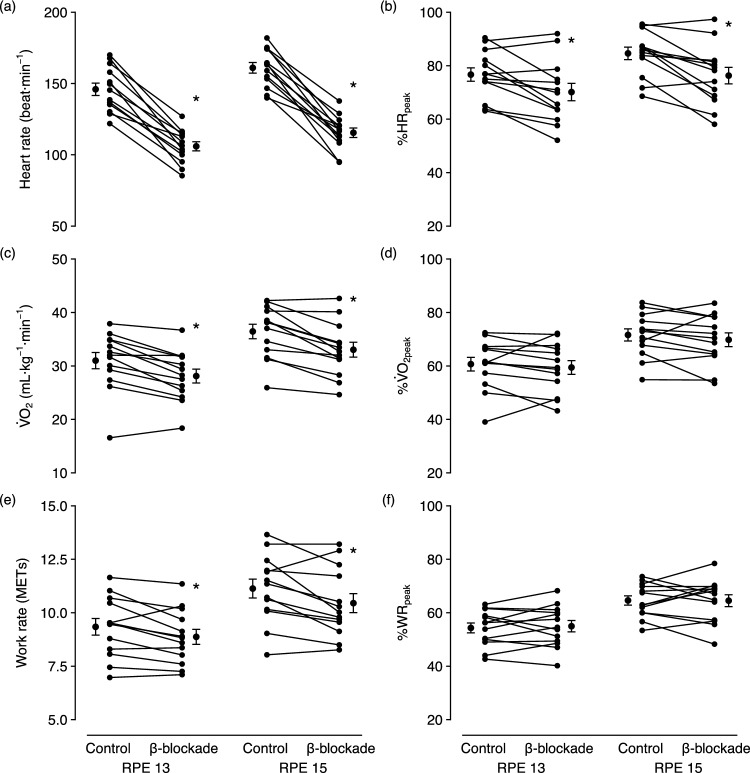
Differences between conditions in individual participant and overall marginal means for heart rate, oxygen uptake and work rate, stratified by RPE level. Error bars represent mean ± SE. *p < 0.05 vs. control

The mean ± SD and range of responses, averaged across bouts for each participant, are summarised in Table 2. Figure 3 illustrates planned contrasts of marginal means between conditions, highlighting the average effect of β-blockade at each RPE level. Contrasts indicated that β-blockade significantly lowered heart rate at both RPE 13 (mean difference [95% CI] = 40.0 beats min^−1^ [36.3, 43.8], *p* < 0.001) and RPE 15 (45.5 beats min^−1^ [41.8, 49.3], *p* < 0.001), with effects remaining significant relative to peak heart rate (RPE 13: 6.53% [4.49, 8.57], *p* < 0.001; RPE 15: 8.32% [6.28, 10.36], *p* < 0.001). Furthermore, ${\dot{\rm{V}}}$O_2_ and work rates were significantly lower during β-blockade at both RPE 13 (2.89 mL kg^−1^ min^−1^ [1.99, 3.80], *p* < 0.001; 0.47 METs [0.21, 0.72], *p* < 0.001) and RPE 15 (3.42 mL kg^−1^ min^−1^ [2.52, 4.32], *p* < 0.001; 0.68 METs [0.43, 0.94], *p* < 0.001), but not when expressed relative to their within-condition peak values (all *p* > 0.05).

**Table 2:** Mean ± SD and range of exercise responses at RPE 13 and RPE 15

	Control		β-Blockade	
	RPE 13	RPE 15	RPE 13	RPE 15
Heart rate, beat min^−1^	146.0 ± 15.7 (121.9–170.0)	161.0 ± 13.4 (139.9–182.0)	106.0 ± 11.6 (85.3–127.0)	115.5 ± 12.1 (94.8–137.7)
%HR_peak_	76.7 ± 9.1 (63.1–90.5)	84.6 ± 8.4 (68.6–95.6)	70.1 ± 11.7 (52.1–92.0)	76.3 ± 11.2 (58.1–97.4)
${\dot{\rm{V}}}$O_2_, mL kg^−1^ min^−1^	31.0 ± 5.5 (16.6–37.9)	36.5 ± 4.9 (25.9–42.2)	36.5 ± 4.9 (25.9–42.2)	33.0 ± 5.0 (24.6–42.6)
%${\dot{\rm{V}}}$O_2peak_	60.7 ± 9.2 (39.1–72.4)	71.6 ± 8.2 (54.9–83.7)	59.4 ± 9.4 (43.2–72.2)	69.8 ± 9.3 (53.5–83.5)
Work rate, METs	9.3 ± 1.4 (7.0–11.6)	11.1 ± 1.6 (8.0–13.7)	8.9 ± 1.3 (7.1–11.4)	10.4 ± 1.6 (8.3–13.2)
%WR_peak_	54.3 ± 6.7 (42.6–63.1)	64.6 ± 6.2 (53.4–73.5)	55.0 ± 7.7 (40.2–68.2)	64.5 ± 8.0 (48.3–78.5)

Values presented as mean ± SD (range) of the average response across bouts for each participant

Intraclass correlation coefficients, presented in Table 3, indicated good to excellent reproducibility of exercise responses across bouts. Although differences between conditions were not statistically significant at either RPE level (all *p* > 0.05), β-blockade generally exhibited slightly wider confidence intervals than control, particularly at RPE 13. Across all outcomes, ICC values tended to be higher with narrower confidence intervals at RPE 15 compared to RPE 13.

**Table 3:** Intraclass correlation coefficients for measures of heart rate, oxygen uptake and work rate across repeated bout of perceptually regulated exercise

	RPE 13			RPE 15		
	Control	β-Blockade	*p*	Control	β-Blockade	*p*
Heart rate	0.75 (0.20, 0.93)	0.79 (0.44, 0.93)	0.807	0.88 (0.63, 0.96)	0.86 (0.59, 0.96)	0.829
%HR_peak_	0.78 (0.24, 0.94)	0.91 (0.68, 0.97)	0.316	0.92 (0.72, 0.98)	0.93 (0.74, 0.98)	0.827
${\dot{\rm{V}}}$O_2_	0.86 (0.70, 0.95)	0.85 (0.49, 0.96)	0.919	0.93 (0.84, 0.98)	0.93 (0.82, 0.98)	0.949
%${\dot{\rm{V}}}$O_2peak_	0.83 (0.63, 0.94)	0.85 (0.44, 0.96)	0.874	0.91 (0.79, 0.97)	0.91 (0.78, 0.97)	0.990
Work rate	0.88 (0.72, 0.96)	0.87 (0.69, 0.95)	0.928	0.96 (0.90, 0.99)	0.96 (0.91, 0.99)	0.859
%WR_peak_	0.83 (0.64, 0.94)	0.88 (0.72, 0.96)	0.651	0.92 (0.81, 0.97)	0.94 (0.86, 0.98)	0.677

Values presented as ICC (95% confidence interval). ICCs calculated via a two-way, mixed-effects model using an absolute agreement definition for single measures. *p*-values derived from *z*-tests following Fisher’s *z*-transformation

Table 4 summarises the CVs for heart rate, ${\dot{\rm{V}}}$O_2_ and work rate across bouts. All individual coefficients were typically below 10%, although this threshold was exceeded once by different participants for heart rate and work rate, and by three participants for ${\dot{\rm{V}}}$O_2_, all during RPE 13. ANOVA showed that variability was significantly lower during exercise at RPE 15 than at RPE 13 across all measures, with no significant differences between control and β-blockade conditions. However, the significant condition × RPE interaction for heart rate highlighted that the reduction in CV from RPE 13 to RPE 15 in the control condition was nearly twice as large as that observed under β-blockade.

**Table 4:** Coefficients of variation (%) in heart rate, oxygen uptake and work rate across repeated bouts of perceptually regulated exercise

	RPE 13		RPE 15		*p*-values		
	Control	β-Blockade	Control	β-Blockade	Con	RPE	Con × RPE
Heart rate	5.5 ± 2.8	4.9 ± 2.2	2.8 ± 1.1	3.5 ± 1.8	0.921	0.003	0.042
${\dot{\rm{V}}}$O_2_	5.8 ± 3.1	5.5 ± 3.2	3.3 ± 1.4	3.7 ± 1.5	0.931	0.004	0.577
Work rate	4.5 ± 2.5	4.2 ± 2.6	2.6 ± 1.6	2.8 ± 1.4	0.969	0.032	0.661

Values presented as mean ± SD of individual participant values. *p*-values for main effects of condition (Con), RPE level (RPE) and their interaction (Con × RPE) derived from repeated measures ANOVA

## Discussion

In this study, we examined the effects of β-blockade on RPE-regulated exercise and the reproducibility of heart rate, ${\dot{\rm{V}}}$O_2_ and work rate across repeated bouts within a single exercise session. As hypothesised, heart rates were consistently lower during β-blockade, with a more pronounced reduction observed at the higher RPE. In contrast to our hypothesis, β-blockade also affected ${\dot{\rm{V}}}$O_2_ and work rate with significant reductions in absolute values at both RPE 13 and RPE 15. Relative to their within-condition peak, neither ${\dot{\rm{V}}}$O_2_ nor work rate different between conditions, aligning with our expectation that β-blockade would not alter relative indices. However, relative heart rate remained significantly lower with β-blockade. High ICCs and low CVs were observed across all measures. Consistent with our hypotheses, coefficients did not significantly differ between conditions, although CVs were consistently lower for the RPE 15 bouts than the RPE 13 bouts.

Building on our previous work, which examined the effect of β-blockade on physiological responses and perceived effort during graded exercise testing (Mitchell et al. 2019), the present study is the first to investigate its impact on physiological and work rate responses across repeated bouts of short-duration, RPE-regulated exercise. Furthermore, to our knowledge, no prior research has examined the intra-session reproducibility of responses to RPE-regulated exercise, with existing studies addressing only inter-session reproducibility. These previous studies predominantly report responses obtained from submaximal perceptually regulated exercise tests or during exercise at RPE levels aligned with specific physiological thresholds. These distinctions limit direct comparisons with the present study as the application of RPE-regulated exercise differs substantially. Nonetheless, values for RPE 13 (50–70%${\dot{\rm{V}}}$O_2peak_) and RPE 15 (60–80%${\dot{\rm{V}}}$O_2peak_) reported in these studies (Eston and Williams 1988; Eston et al. 2005, 2012; Faulkner et al. 2007; Morris et al. 2009, 2010; O’Grady et al. 2021) align with those observed here, where participants exercised at approximately 60% and 70%${\dot{\rm{V}}}$O_2peak_ for RPE 13 and RPE 15, respectively, during both control and β-blockade conditions (Table 2). Work rates were typically 55% and 65%WR_peak_ for RPE 13 and RPE 15, respectively, while relative heart rates were slightly lower with β-blockade, averaging 70% and 75%HR_peak_ for RPE 13 and RPE 15, compared to 75% and 85%HR_peak_ during control conditions (Table 2). Relative work rate and heart rate values were not reported in the earlier studies.

The consistency of participant responses across exercise bouts was a central focus of this study. No main or interaction effects were observed for exercise bout, suggesting that participants selected similar work rates, eliciting comparable oxygen uptake requirements across bouts for each RPE level. These consistent responses were evident across both conditions, indicating that β-blockade did not affect the reproducibility of exercise intensities. Absolute and relative measures of heart rate did increase across bouts, however the absence of a significant RPE level × bout interaction suggests this increase was more likely due to cardiovascular drift than an impaired ability to self-regulate exercise intensity at the prescribed RPE levels.

The high reproducibility of individual participant exercise responses across bouts was further validated by the ICC and CV analyses. For both the absolute and relative metrics of ${\dot{\rm{V}}}$O_2_ and work rate, ICCs were consistently high, and CVs consistently low, with no significant difference between conditions. These findings emphasise the potential for RPE-regulated exercise to facilitate highly reproducible exercise responses across bouts, particularly at higher exercise intensities, and underscore the need to consider individual variability when prescribing exercise at lower RPE levels.

Reduced heart rates during exercise are a well-established consequence of β-blockade. In this study, we observed average reductions of 40 beat min^−1^ across the RPE 13 bouts and 45.5 beat min^−1^ during RPE 15. These corresponded to 27.3 and 28.3% reduction over the control condition, respectively, aligning with the expected range of 15–30% observed in previous studies (Van Baak 1988; Eston and Connolly 1996). Most prior research has focused on the effects of β-blockade on exercise heart rates at either fixed workloads or standardised work rates set externally to the individual (Ekblom and Goldbarg 1971; Davies and Sargeant 1979; Myers et al. 1987; Mitchell et al. 2019). In contrast, our study allowed participants to select work rates based on their perceived exertion, extending previous findings by demonstrating comparable reductions in heart rate with β-blockade during perceptually regulated exercise.

Despite studies reporting strong linear associations between heart rate and RPE (Borg and Linderholm 1967; Chen et al. 2002), our findings suggest that heart rate is not a major contributor to the perceived exertion gestalt. If heart rate were a primary mediator of perceived exertion, when permitted to self-regulate exercise intensity using RPE, we would expect participants to select work rates that elicit similar heart rates during β-blockade and control. Our finding that differences in heart rate between conditions remained significant when standardised to %HR_peak_ further supports the hypothesis that heart rate does not directly drive the perception of effort.

The significant reductions in work rate and ${\dot{\rm{V}}}$O_2_ with β-blockade did not support our original hypothesis. Work rates were typically 0.5 METs lower for RPE 13 and 0.7 METs for RPE 15 and suggests participants perceived exercise to be harder—or more intense—when performed under β-blockade. Since participants regulated their own intensity, the perceived increase in effort manifested in selecting lower work rates to maintain the target RPE. This likely contributed to the reductions in ${\dot{\rm{V}}}$O_2_ of 2.89 mL kg^−1^ min^−1^ for RPE 13 and 3.42 mL kg^−1^ min^−1^ for RPE 15 observed with β-blockade. It remains unclear, however, whether the observed reduction in work rate drives the reduction in exercise ${\dot{\rm{V}}}$O_2_ or reflects a compensatory response to the reduced ${\dot{\rm{V}}}$O_2_ itself. Nevertheless, the lower work rates are unlikely to account for all the decrease in ${\dot{\rm{V}}}$O_2_, as the reductions in absolute work rate corresponded to 5.4 and 6.3% relative to control for RPE 13 and RPE 15, respectively, while absolute ${\dot{\rm{V}}}$O_2_ decreased by 9.3% for both RPE levels. These results highlight the distinct metabolic effects of β-blockade on exercise ${\dot{\rm{V}}}$O_2_ that extend beyond the reductions attributable solely to the decrease in selected work rates.

A key strength of this study was the robust placebo-controlled, counter-balanced design, allowing for within-subject comparisons of the effects of β-blockade and exercise responses. Despite this, several limitations should be acknowledged. The acute administration of β-blockade, while effective in eliciting the intended cardiovascular effects, may not fully replicate the physiological and perceptual responses seen with longer-term therapeutic use. Chronic β-blockade may yield distinct physiological and, by extension, perceptual responses that were beyond the scope of this study. However, previous studies with longer-duration exposure to β-blockade have reported conflicting findings. For example, where some studies (Sklar et al. 1982; Wilmore et al. 1985; Wonisch et al. 2003) report no effect of chronic β-blockade on ${\dot{\rm{V}}}$O_2max_, other studies (Anderson et al. 1985; Jilka et al. 1988; Joyner et al. 1986, 1987; Kalis et al. 1988; Tesch and Kaiser 1983) report persistent reductions in ${\dot{\rm{V}}}$O_2max_ consistent with those observed with acute β-blockade here and in other studies (Hughson and MacFarlane 1981; Dodd et al. 1988; Schneider et al. 1994; Wallen et al. 2017). Nevertheless, these findings provide important insights into the acute effects of β-blockade at its onset and further advances our understanding of the physiological basis of effort perception. Future studies are needed to compare these findings with responses following longer-duration exposure to β-blockade.

The study sample consisted of younger, healthier participants with relatively high cardiorespiratory fitness, who were screened to exclude those with clinical conditions. While this ensured safety and minimised any confounding effects of comorbid conditions, it limits the generalisability of these findings to populations typically prescribed β-blockade. These individuals may exhibit distinct physiological and perceptual responses during exercise, highlighting the need for further research focusing on clinical populations to better understand the utility and reproducibility of perceptually regulated exercise in these groups.

Additionally, data were collected from a single exercise session per condition, limiting the interpretation of findings to within-session reproducibility. Normal between-session variability in perceptually regulated exercise could not be accounted for, meaning differences between conditions observed here may partly reflect this variability. Caution should be taken when generalising these results to multiple training sessions or longitudinal exercise programmes without further investigation.

Although the study employed a double-blind design to minimise bias, the pronounced effects of β-blockade on heart rate could have inadvertently allowed research personnel to infer the condition during testing. Nonetheless, the absence of participant encouragement, concealment of real-time physiological and performance data, and withholding of performance feedback until study completion from participants reduced the likelihood of this influencing the results. Future research should consider strategies to enhance blinding where overt physiological responses are involved.

## Conclusion

Our findings support the use of RPE to prescribe and regulate exercise intensity during training, even in the presence of β-blockade. While β-blockade significantly altered the relationship between RPE and heart rate, the association between RPE and ${\dot{\rm{V}}}$O_2_ remained stable, reinforcing the reliability of RPE-regulated exercise when heart rate is less predictable. These findings are encouraging for clinical applications, such as cardiac rehabilitation or heart failure, where β-blocker use is common. Caution is warranted in extending these findings to clinical populations, who typically present with lower fitness, cardiovascular disease, and other age-related complexities not represented in our sample. Clinicians implementing RPE-regulated exercise should first establish target intensities using an RPE-regulated exercise test (e.g., the Perceptually Regulated Exercise Test; Eston et al. 2012), and observe each patient’s ability to regulate intensity over multiple sessions before transitioning to unsupervised training. RPE-regulated exercise may also be less sensitive to changes in medication regimen, potentially reducing the need for repeat exercise testing to adjust intensity prescriptions. Further research is warranted to evaluate the application of RPE-regulated exercise in clinical populations where medication use, comorbidities, and functional limitations may influence effort perception.

## Supplementary Information

Below is the link to the electronic supplementary material.

Supplementary file1 (PDF 199 KB)

## Data Availability

Data are available from the corresponding author upon reasonable request. Code used for statistical analyses and visualisations are available in the GitHub repository: https://github.com/drblmitchell/2024_BetaPRET_RPEIntervals.

## Abbreviations

ACSM: American College of Sports Medicine
ANOVA: Analysis of variance
CV: Coefficient of variation
GXT: Graded exercise test
HR_peak_: Peak heart rate
ICC: Intraclass correlation coefficient
MET: Metabolic equivalent
RPE: Rating of perceived exertion
$${\dot{\rm{V}}}$$O_2_: Oxygen uptake
${\dot{\rm{V}}}$O_2peak_: Peak oxygen uptake
WR_peak_: Peak work rate
