# Supplementary material for: Effect of β-blockade on measures and reproducibility of heart rate, oxygen uptake and work rate across repeated bouts of short-duration, RPE-regulated exercise
Source: Eur J Appl Physiol. 2025 Jun 1;125(11):3247–57. doi: 10.1007/s00421-025-05818-x (PMC12528277; doi:10.1007/s00421-025-05818-x)
Supplement: Supplementary file 1 — Supplementary file1 (PDF 199 KB) [file 421_2025_5818_MOESM1_ESM.pdf]

ONLINE SUPPLEMENTARY MATERIAL

Effect of β-blockade on measures and reproducibility of heart rate, oxygen uptake and work rate across repeated bouts of short-duration, RPE-regulated exercise

Braden L. Mitchell[1] (0000-0002-8091-2549), Kade Davison[1] (0000-0002-7101-7729), Gaynor Parfitt[1] (0000-0002-5547-5797), Roger G. Eston[1] (0000-0002-2680-7473)

[1] Alliance for Research on Exercise Nutrition and Activity (ARENA), University of South Australia, Adelaide SA, Australia

Correspondence

Braden L. Mitchell, Ph.D. (drblmitchell@gmail.com)

SCRIPTED INSTRUCTIONS FOR RPE ANCHORING

While exercising, we want you to rate your perception of effort, that is, how hard and strenuous the exercise feels for you. The perception of exertion depends mainly on the strain and fatigue in your muscles and on your feelings of breathlessness or aches in the chest. It is important that you consider all these exercise sensations as an overall perception and not concentrate on any one sensation individually. Look at this scale [show Borg 6-20 RPE scale]; we want you to use this scale from 6 to 20, where 6 means 'no exertion at all' and 20 means 'maximal exertion'. A 9 on this scale corresponds to 'very light' exercise. For a normal, healthy person it is like walking slowly at your own pace for a few minutes. A 13 on the scale is 'somewhat hard' exercise, but it still feels okay to continue. A rating of 15 represents a hard effort. At this intensity a healthy person is working at a level that requires some effort to maintain but can still continue for a prolonged period if necessary. Breathing is deeper and their muscles feel as though they are working hard, but they are not yet approaching exhaustion. A 17 is 'very hard' and very strenuous exercise. A healthy person can still go on but must really push themselves to continue. It feels very heavy, and the person is very tired. Lastly, a 19 on the scale is an extremely strenuous exercise level. For most people, this is the most strenuous exercise they have ever experienced.

During this session, I am going to ask you to exercise at a 13 and 15 on this scale. I want you to use the scale to help you guide the intensity of the exercise to match what you feel a 13 or 15 is. You are free to adjust the speed and/or incline of the treadmill at any time so that you are exercising at that level. It is important that you maintain an intensity you perceive to be a 13 or 15. If this means you need to adjust the treadmill up or down as the exercise continues that is okay and expected. Take no notice of the treadmill settings, it is your feeling of effort and exercise that is important. Please be aware that I do not have any expectations about your performance and remember that my main interest is that you use your own feelings to control the exercise intensity. Do you have any questions?

SUPPLEMENTARY RESULTS: FULL MODEL OUTPUTS AND DIAGNOSTICS

Table 1 Results of the linear mixed effects model for heart rate

	β	95% CI	t	*p*
Condition	-36.49	(-43.79, -29.20)	-9.66	< .001
RPE level	19.94	(12.64, 27.23)	5.28	< .001
Bout	6.54	(3.80, 9.27)	4.62	< .001
Condition × RPE level	-5.52	(-10.68, -0.36)	-2.07	.041
Condition × bout	-1.76	(-4.92, 1.40)	-1.08	.283
RPE level × bout	-2.44	(-5.60, 0.72)	-1.49	.139

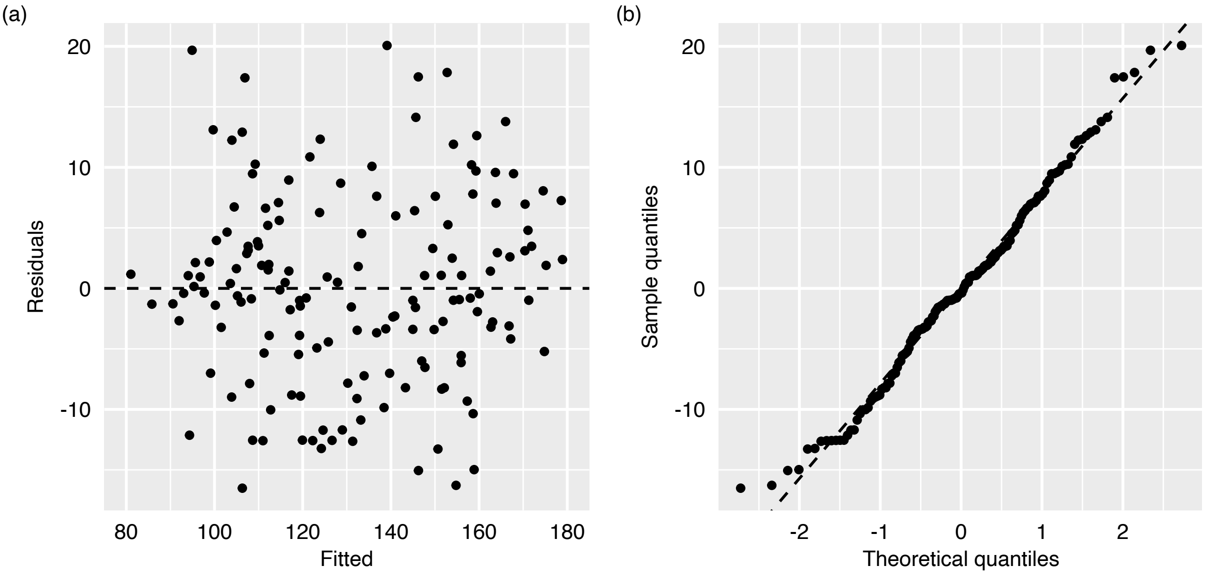

Figure 1 Diagnostic plots for the linear mixed effects model for heart rate: (a) residuals plot and (b) Q-Q plot of residuals

Table 2 Results of the linear mixed effects model for %HR$_{peak}$

	β	95% CI	t	*p*
Condition	-5.55	(-9.54, - 1.56)	-2.69	.008
RPE level	10.53	(6.54, 14.52)	5.10	< .001
Bout	3.45	(1.95, 4.94)	4.45	< .001
Condition × RPE level	-1.79	(-4.60, 1.03)	-1.22	.223
Condition × bout	-0.49	(-2.22, 1.23)	-0.55	.583
RPE level × bout	-1.30	(-3.02, 0.43)	-1.45	.149

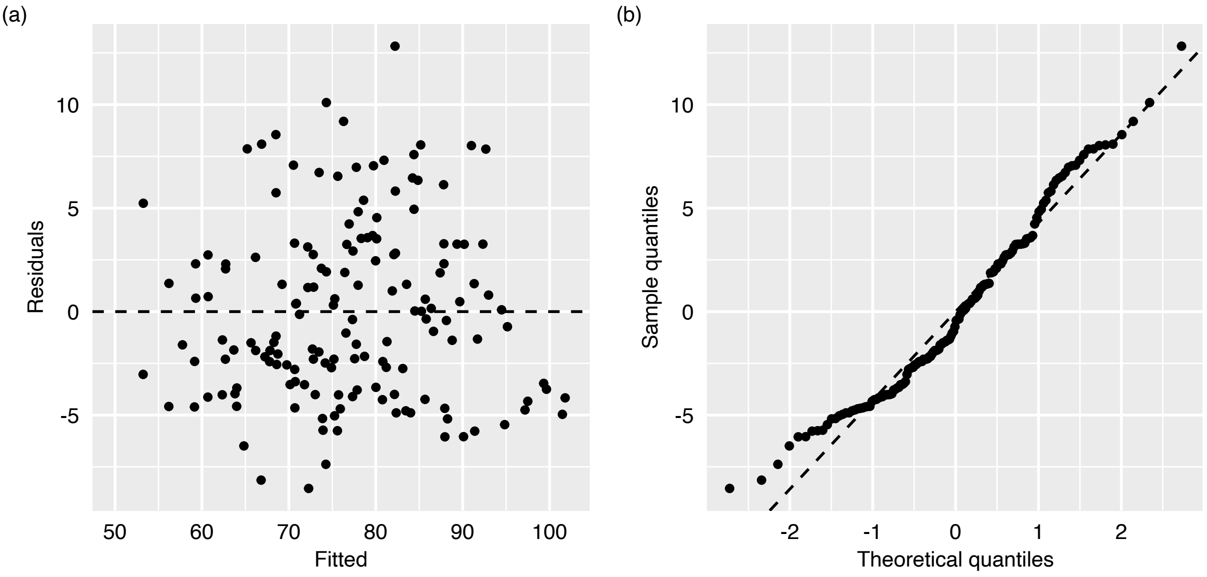

Figure 2 Diagnostic plots for the linear mixed effects model for %HR$_{peak}$: (a) residuals plot and (b) Q-Q plot of residuals

Table 3 Results of the linear mixed effects models for oxygen uptake

	β	95% CI	t	*p*
Condition	-4.22	(-5.99, -2.46)	-4.62	< .001
RPE level	6.55	(4.79, 8.32)	7.16	< .001
Bout	0.54	(-0.12, 1.20)	1.58	.116
Condition × RPE level	-0.53	(-1.78, 0.72)	-0.82	.416
Condition × bout	0.67	(-0.10, 1.43)	1.68	.095
RPE level × bout	-0.55	(-1.32, 0.22)	-1.39	.167

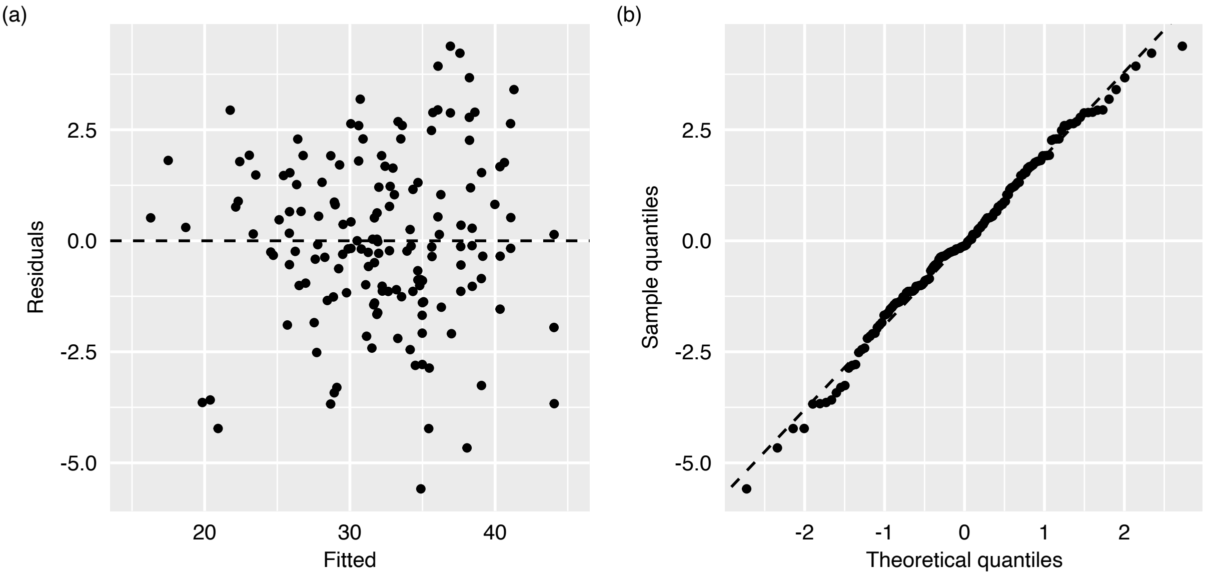

Figure 3 Diagnostic plots for the linear mixed effects model for oxygen uptake: (a) residuals plot and (b) Q-Q plot of residuals

Table 4 Results of the linear mixed effects model for $\%\dot{V}O_{2peak}$

	β	95% CI	t	*p*
Condition	-4.07	(-7.84, -0.30)	-2.09	.039
RPE level	13.24	(9.48, 17.01)	6.79	< .001
Bout	1.14	(-0.27, 2.55)	1.56	.121
Condition × RPE level	-0.54	(-3.21, 2.12)	-0.40	.694
Condition × bout	1.41	(-0.22, 3.04)	1.67	.098
RPE level × bout	-1.16	(-2.79, 0.47)	-1.38	.171

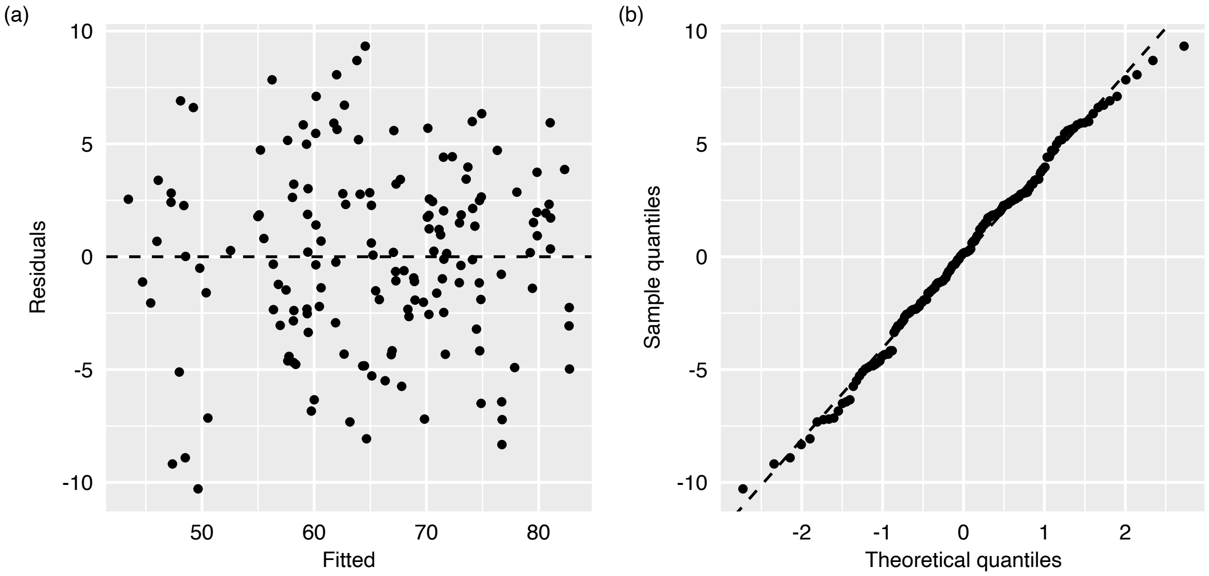

Figure 4 Diagnostic plots for the linear mixed effects model for $\%\dot{V}O_{2peak}$: (a) residuals plot and (b) Q-Q plot of residuals

Table 5 Results of the linear mixed effects model for work rate

	β	95% CI	t	*p*
Condition	-0.60	(-1.10, -0.10)	-2.32	.022
RPE level	2.22	(1.72, 2.72)	8.59	< .001
Bout	0.15	(-0.04, 0.33)	1.51	.133
Condition × RPE level	-0.21	(-0.57, 0.14)	-1.17	.244
Condition × bout	0.07	(-0.15, 0.28)	0.59	.558
RPE level × bout	-0.22	(-0.43, 0.00)	-1.93	.056

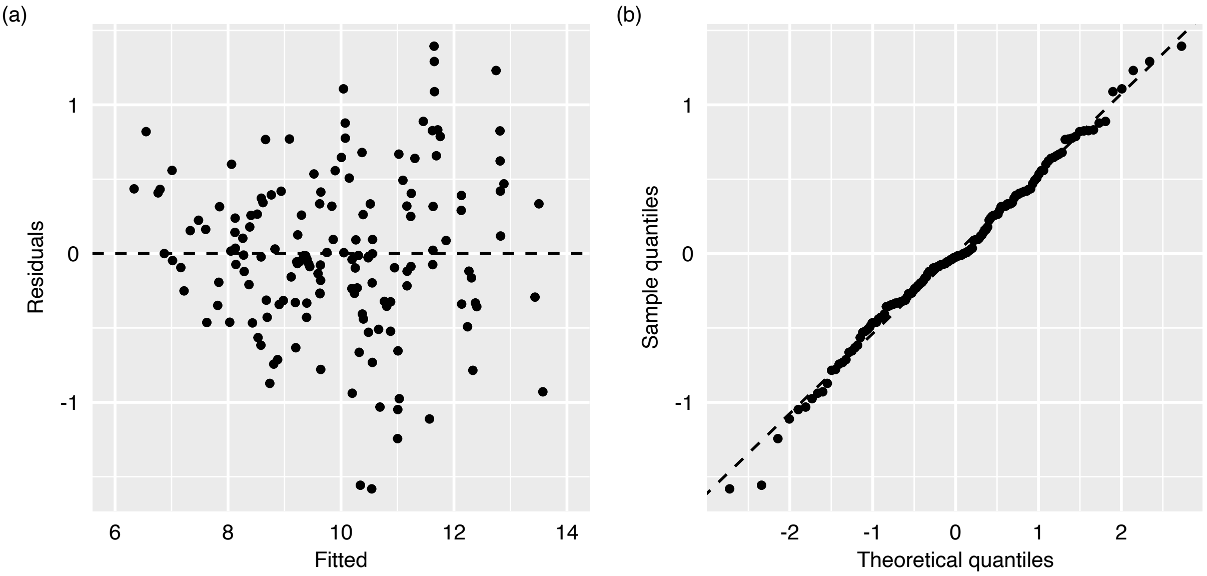

Figure 5 Diagnostic plots for the linear mixed effects model for work rate: (a) residuals plot and (b) Q-Q plot of residuals

Table 6 Results of the linear mixed effects model for %WR_{peak}

	β	95% CI	t	*p*
Condition	-0.15	(-3.19, 2.89)	-0.10	.924
RPE level	12.86	(9.82, 15.90)	8.17	< .001
Bout	0.87	(-0.27, 2.01)	1.47	.144
Condition × RPE level	-0.72	(-2.87, 1.44)	-0.64	.522
Condition × bout	0.39	(-0.92, 1.71)	0.58	.565
RPE level × bout	-1.29	(-2.61, 0.02)	-1.90	.060

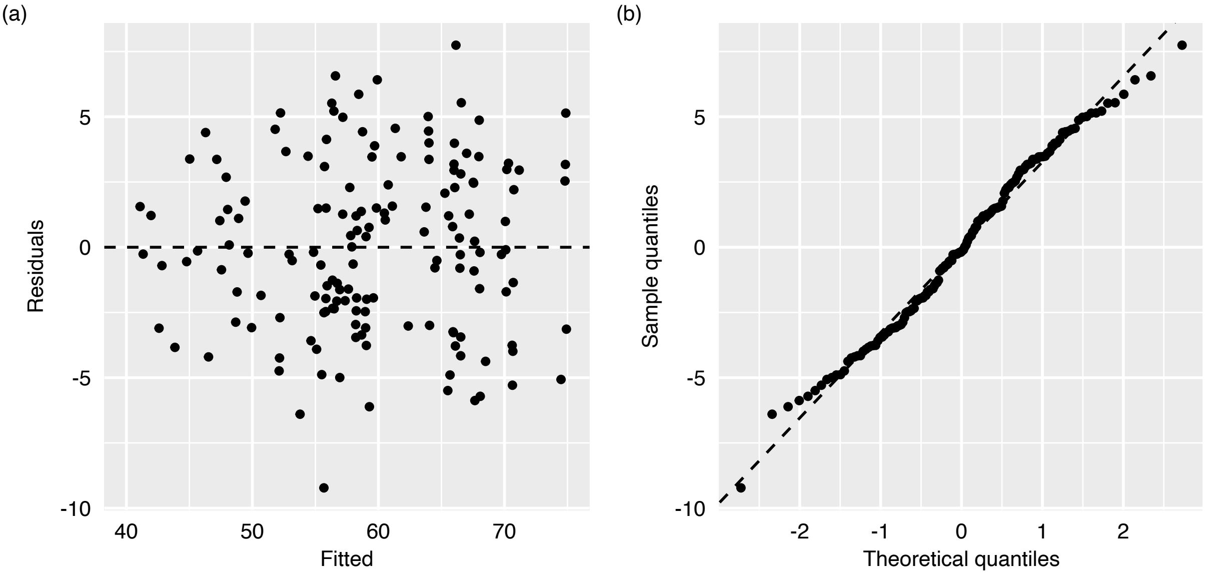

Figure 6 Diagnostic plots for the linear mixed effects model for %WR_{peak}: (a) residuals plot and (b) Q-Q plot of residuals
